# Patients’ experiences of ward rounds in a Swedish context: a qualitative study

**DOI:** 10.1136/bmjopen-2025-103481

**Published:** 2025-11-13

**Authors:** Emma Larsson, Yelyzaveta Hordiienko, Cecilia Fagerström, Sofia Almerud-Österberg, Hafrún Rafnar Finnbogadóttir, Carina Persson

**Affiliations:** 1 Department of Oncology Clinic and Radiation Physics, Region Kalmar County, Kalmar, Sweden; 2 Department of Health and Caring Sciences, Linnaeus University, Kalmar, Sweden; 3 Department of Research, Region Kalmar County, Kalmar, Sweden; 4 Department of Anaesthesiology, Region Kronoberg, Växjö, Sweden

**Keywords:** Patient Participation, Inpatients, Patient-Centered Care, QUALITATIVE RESEARCH, Patients

## Abstract

**Abstract:**

**Objective:**

The aim was to explore patients’ experiences of ward rounds in inpatient care.

**Design:**

An exploratory qualitative design was chosen, collecting data with one-to-one semistructured interviews, conducted from March to May 2023. An interview guide was used as a basis for the interviews. Data were analysed using reflexive thematic analysis.

**Setting:**

A medical and a surgical ward at a medium-sized hospital in southern Sweden.

**Participants:**

Purposeful sampling was used to recruit patients aged 18 years or older. 16 patients were recruited with an age range of 38–72 years.

**Results:**

The findings showed that patients’ experiences of ward rounds have a wide range of variation. The main theme was: ‘The ward round as a bridge between patients’ experiences and knowledge and healthcare professionals’. The main theme consisted of two subthemes, reflecting the variation in patients’ experiences: ‘Feeling of togetherness versus loneliness’ and ‘Getting answers or being left in limbo’. The subthemes also highlighted patients’ experiences of factors that enabled satisfactory interactions between patients and healthcare professionals during ward rounds, such as comprehensible detailed information and supportive atmosphere, as well as experiences of factors that obstructed such interactions and caused patients to feel uncertainty.

**Conclusions:**

Ward rounds in inpatient care play an important role for patient care and health, functioning as a bridge between patients and healthcare professionals. It is important for healthcare practitioners and policy-makers to create a model for ward rounds that can contribute to an open and supportive atmosphere as well as sharing comprehensible and detailed information.

**STRENGTHS AND LIMITATIONS OF THIS STUDY**

- The two first authors wrote reflexive journals, which promoted their reflexivity and revealed their preunderstandings.
- All the authors continuously discussed their preunderstandings and reflected on the analysis to uncover any values and assumptions taken for granted.
- Transferability and confirmability were ensured through thick descriptions of the study population and context as well as data collection and analysis.
- Gatekeepers’ help in patients’ recruitment and patients’ reluctance to criticise rounds may influence the result of the study.
- The sample was Swedish-speaking patients from a single hospital, which may limit transferability.

## Introduction

 The ward round is an important part of daily clinical work in inpatient care, enabling interaction between patients and healthcare staff.^1^ The purpose of the ward round is to plan medical and nursing care and gather information through communication between patients and healthcare professionals.^2^ It can be an opportunity to involve a patient in decisions about their healthcare, to ensure that it meets their needs and preferences. Giving the patient the opportunity to share their experiences with healthcare professionals and involving them in decision-making regarding their own care can contribute to increased patient satisfaction, quality of care and patient safety.^3 4^

There are different sorts of ward rounds: consultant, teaching, multidisciplinary, sitting, walking and ‘post-take’.^5^ Only the ward rounds with a multidisciplinary team involve patients in treatment and discharge plans discussions, while other types of rounds, for example, consultant and teaching rounds, focus on communication between healthcare professionals who discuss patient care, which may influence patients’ experiences.^5^

Previous studies show that patients feel excluded from the discussions that take place during rounds^3 6^ and are not seen as individuals.^6^ Patients have described the traditional procedure of rounds as a parade led by a senior doctor followed by junior doctors, medical students and nursing staff focusing on the doctor’s priorities—diagnosis, physical findings and teaching.^1^ It also appears, according to the Australian study, that patients feel uncertain about their role during rounds and that their experiences of ward rounds are important for their commitment, expectations and opportunity to participate.^7^ These experiences concern multidisciplinary rounds, where several professions participate, which are in focus of the present study.

Patients’ definition of patient participation includes acquiring and being able to apply knowledge about disease, symptoms and treatment, as well as sharing experiences and knowledge about disease and treatment.^8^ Interactions with the healthcare professionals, meaning sharing the same care aims, respecting each other for knowledge and mutual contribution to communication, are often emphasised rather than participation in decision-making.^9^ Having knowledge is emphasised rather than being informed, meaning that comprehending information and knowing where to turn for help and what to do to get well is more important for patients than receiving personally tailored information.^9^ However, it appears that the meaning of patient participation in clinical practice is often limited to participation in decisions about care and treatment and that few rounds allow for patient contributions in decisions about care.^4 6^ In a previous study, it appeared that patients’ preferred roles in medical decision-making, their decision control preferences (DCPs), differ and are not associated with a patient’s perceived medical knowledge.^10^ Some patients prefer to leave decision-making to the healthcare professional (passive DCP), some prefer to choose treatment options themselves (active DCP), and some prefer sharing decision-making with the healthcare professionals (collaborative DCP). Active DCP is associated with lower satisfaction with the care provided and lower trust in the care team. Having knowledge of a patient’s preferences is therefore important to enable personalised patient-centred care.^11^

Patient-centred care means that the patient’s wishes and needs should be the starting point for care, and that care and self-care should be based on active patient participation. A central aspect is that patient participation must be voluntary and based on the patient’s wishes and needs. To achieve patient participation, healthcare professionals must relinquish some of their power and there must be a relationship between care provider and patient. Active and mutual engagement in intellectual and/or physical activity as well as shared knowledge and information are central aspects.^12 13^

A systematic review aimed to explore the available evidence on the performance of rounds, differences in definitions and effects of rounds on team collaboration, care quality and patient-centred care.^14^ The review highlighted the lack of European research. The healthcare system in Europe has strong primary care and universal coverage, while, for example, the Australian healthcare system relies both on public and private hospitals.^15 16^ The mentioned aspects of the European healthcare system may have an impact on the ward round organisation and, therefore, on patients’ experiences of the rounds. In addition, patient-centredness was identified as a key aspect of rounds that required further exploration.^14^ Furthermore, when patients are engaged in their care, it contributes to their positive health outcomes, positive experiences of care and safety.^17 18^ Increased knowledge about the patient experience of rounds is thus important for the healthcare professionals involved, to be able to improve the patient-healthcare relationship and patient satisfaction. This can contribute to improving quality and safe care that is based on each individual patient’s needs and goals.

## Aim

The aim was to explore patients’ experiences of ward rounds in inpatient care.

## Methods

### Design

An exploratory qualitative design was chosen to explore patients’ experiences of ward rounds using semistructured interviews^19 20^ and reflexive thematic analysis.^21^ Reflexive thematic analysis is a method for active and careful developing, analysing and reporting themes within qualitative data.

### Participants/sampling

A purposeful sampling was used to capture information-rich cases for exploration of patients’ experiences of ward rounds.^22^ In the selection of patients, as wide a spread as possible was sought regarding age, gender and healthcare experience.

To be offered participation in the study, the patient had to be aged 18 years or older, admitted to one of the relevant wards, have experience of at least one round during the current care session, and speak and understand the Swedish language well enough to be able to give informed consent and participate in an interview. Patients who were not in the physical or mental condition to absorb information and give informed consent to participate were not asked. Identification of patients who, based on the criteria, could be asked about participation was performed with the help of a local contact person, a gatekeeper, in each relevant department. The gatekeepers were patient care coordinating nurses, who have the continuous and overall responsibility of a separate patient care, its planning, conducting, assessment and evaluation.

In total, 36 participants were asked to participate in the study. 16 patients (9 women and 7 men) accepted the invitation. The median age was 72 years (range 38–83 years). Other patients declined the participation due to their health state or unwillingness.

### Data collection and settings

The setting was a medical and a surgical ward at a medium-sized hospital in southern Sweden, where data were collected from March to May 2023. Inpatient care was provided, where patients were admitted and remained in the hospital for treatment and examination. The medical ward had 28 beds, divided across four care teams with seven patients and one nurse, one doctor and one junior doctor per care team. At the surgical ward, there were 21 beds, with each nurse responsible for up to 7 patients and each doctor responsible for 0–14 patients, depending on the patient category. The procedures for rounds were similar on the selected wards. First, the doctor and nurse in charge of a patient discussed that patient’s situation and status. Only rarely was the assistant nurse present on such occasions, due to lack of time. After this, the doctor went to the patient’s room; the nurse in charge attended if she had time. The time for rounds varied, but they usually started between 8:30 and 10:00.

An interview guide was used as a basis for the interviews, containing questions aimed to explore the patient’s perspective of rounds, regarding both the round procedure and its contents, and their own role, the interaction and what they considered to be the purpose of the rounds (online supplemental file 1). While developing the interview guide, the researchers were focusing on categories of patient-centred care defined by patients, such as involvement in treatment, information provided, partnership, respectful treatment, communication, patient as priority, accessible care.^11^ The questions in the interview guide included (online supplemental file 1): *Could you tell me about how rounds are performed here at the ward? How do you see your own role and participation in rounds? What do you think is important to address during rounds? Can you tell us about a positive round situation?/Is there any round that you have experienced particularly well? How would you like rounds to be performed?* Open-ended questions allowed the participants to talk freely about their experiences. With these questions, patients’ DCPs could be captured, though no direct questions were created aiming to this. The interviewer was free to ask follow-up questions if necessary to gain a deeper understanding of what was described during the conversation.

The interviews were conducted by two research nurses separately (each conducted eight interviews) who did not work at the selected wards and had no relations to the participants; still, they had knowledge of how the rounds were conducted from the communication with colleagues and gatekeepers. Furthermore, they got information about the structure of the rounds from patients during interviews. A pilot interview was conducted with a patient who met the criteria for inclusion in the study. This interview, which was not included in the analysis, was performed in part to test the interview guide and identify any shortcomings, and in part so the interviewers would have the opportunity to uncover any weaknesses in their own performance. The patient felt that the questions were relevant, contributed to reflection and made it possible to describe their experiences. The interview guide was therefore used without any changes. Each interview was conducted under calm conditions at the time and place chosen by the participant. Most interviews were completed face-to-face at the wards, but two interviews were conducted by telephone due to the participants being discharged. The final two interviews gave no new information and data collection ended. The interviews ranged between 9 and 36 min (median 15 min). The interviews were recorded to a separate device and later transcribed into text for further analysis using reflexive thematic analysis.

### Data analysis

**Figure 1:**
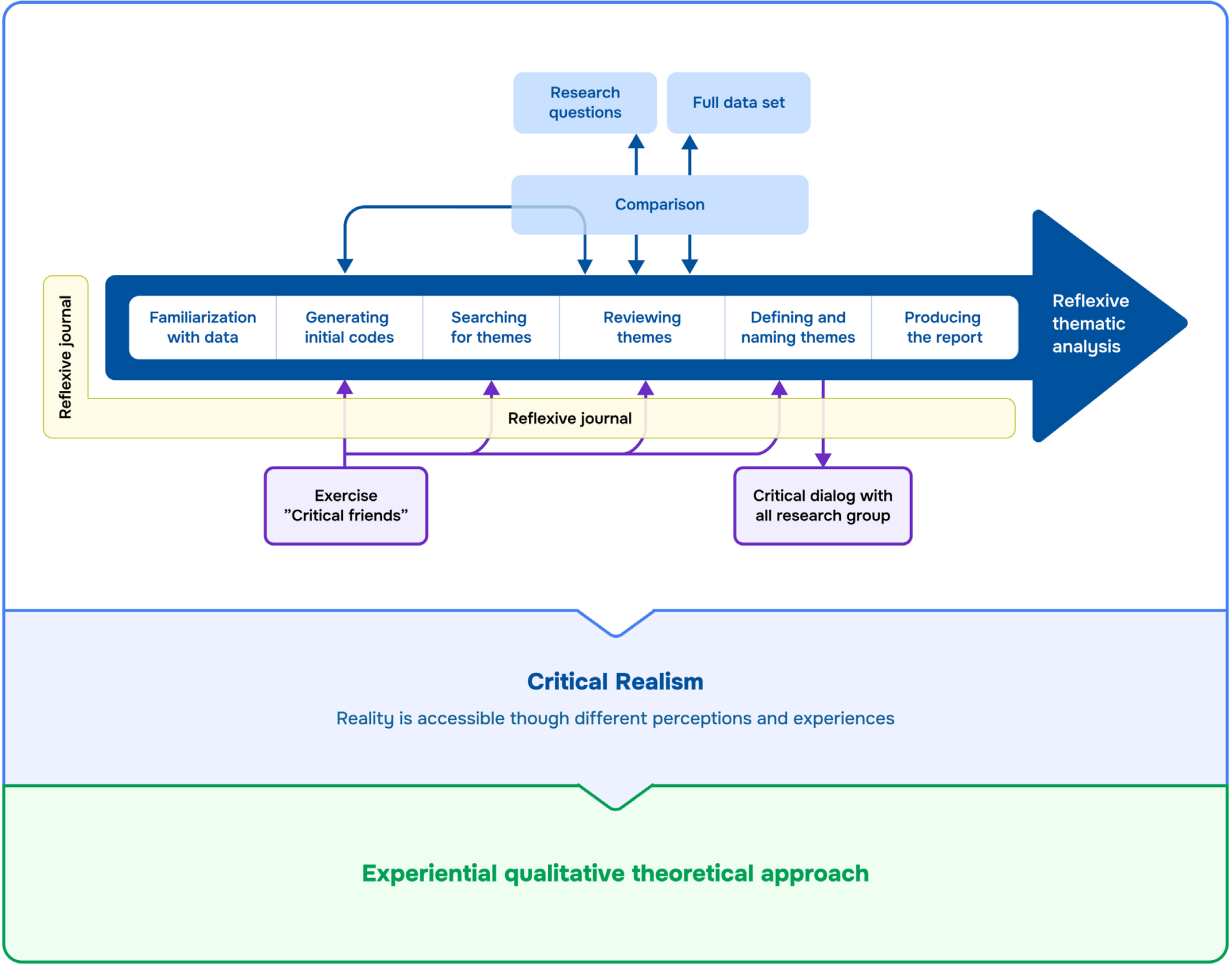
Phases of reflexive thematic analysis inspired by Braun and Clarke.^21^

Transcribed data were analysed using reflexive thematic analysis to develop themes within the dataset.^21^ An experiential qualitative theoretical approach was used for the analysis, as data were based on patients’ experiences of ward rounds, where language is viewed as a means of looking into patients’ realities and meanings.^21^ The analysis was informed by critical realism, according to which reality is socially dependent and can be interpreted and conceptualised in different ways.^21^ Thus, the researchers and informants were believed to influence each other during data collection and analysis, and it was important for the researchers to discuss their interpretations of patients’ experiences of ward rounds, acknowledging each other’s subjectivity and challenging preunderstandings. The analytical process underwent the following phases (figure 1).

The data were carefully read through several times by the first authors (EL and YH) in an active, analytical and critical way. Reactions and ideas, related to both specific data items and the dataset as a whole, were noted and discussed. Data relevant to the purpose of the study were identified and marked by the first authors separately and systematically, with a focus on identifying data and creating codes that captured the underlying meaning of what was said. Codes were then discussed and compared between the first two authors until consensus was reached. Coded data were carefully reviewed and codes with similar meaning formed potential themes. The potential themes were reviewed in relation to the research question by the first two authors and the rest of the research group and analysed to check their consistency with the extracted codes and the full dataset. The potential themes were further analysed and refined to explore the specifics of each theme. This process aimed to identify clear and well-defined names for each theme.^21^ The analysis was discussed with the research group in a critical dialogue, to promote reflexivity and challenge each other’s interpretations. Guidance from the research group was significant in proving that subjectivity was acknowledged and a prerequisite for generating a careful interpretation and analysis of the data. This confirmed that theoretical assumptions about the concept of patient participation were active components of the analysis, though the authors promoted multiple interpretations of the data and revealed their preunderstandings. The patients’ quotations were translated from Swedish into English by the first authors and the translation was reviewed by an authorised language reviewer. Table 1 shows an excerpt of the analysis process.

**Table 1:** An example of the analysis process according to Braun and Clarke^21^

Compressed data extracts	Codes	Subthemes	Theme
The doctor chose his words carefully and expressed himself correctly. The doctor asked if I followed along and understood the whole thing and I did (*patient 2*). The doctor explains test results, so I understand (*patient 12*). I feel well treated and that the doctor is factual and clear in his information (*patient 16*).	Getting comprehensible and detailed information	Getting answers or being left in limbo	The ward round as a bridge between patients’ experiences and knowledge and healthcare professionals
I am the recipient of information and can only ask counter-questions (*patient 11*). I feel like a package lying in bed and can just say thank you and accept what is done. The round is primarily about the doctor giving the patient information (*patient 1*). They come in, introduce themselves, half of them ask how the patient is doing, some of them just do their thing—this is how we do it and leave. I know my body, but many doctors don't listen unless I get angry (*patient 10*).	One-way communication		
I felt involved and felt that I could take the space I wanted during rounds (*patient 8*). I get space to talk and answer questions about how I experience it all (*patient 6*). The doctor conducts the conversation but asks me questions. The nurse asks about my experience (*patient 7*).	Being invited into the dialogue and get space	Feeling of togetherness vs loneliness	
The doctor leaned against the toilet door and said, ‘you should know that you have a cancerous tumour’. The doctor’s manner and choice of words made me sad and shocked (*patient 2*). The doctor can feel distant (*patient 11*). At one point, the doctor barely looked me in the eye and spoke to the ‘entourage’, not to me. I told the doctor, who was shocked, but sat on the edge of the bed (*patient 13*).	Not being treated professionally		

### Reflexivity

EL is a registered specialist nurse with extensive patient care experience. YH has a pedagogical education and a master’s degree in health science. Their different backgrounds and education as well as absence of power differentials served as a good basis for open discussions and challenging each other’s perspectives on the data. Both wrote reflexive journals, which enhanced the trustworthiness of the study. CF is a researcher at the regional research department and a professor of caring science. SAÖ is a clinical lecturer and associate professor of caring science. HRF is an associate professor of caring science. CF and HRF are registered nurses, and HRF is also a registered midwife. Both have experience of ward rounds in inpatient care from various departments and settings. CP is a registered physiotherapist and associate professor of caring science, with extensive experience of qualitative research. The differing backgrounds and substantive experiences of the authors contributed to enriching the discussions and reflections within the study.

## Findings

One main theme was generated from the empirical data: ‘The ward round as a bridge between patients’ experiences and knowledge and healthcare professionals’. The main theme consisted of two subthemes: ‘Feeling of togetherness vs loneliness’ and ‘Getting answers or being left in limbo’. The subthemes include codes highlighting patients’ experiences of factors that enable interaction between patients and healthcare professionals that can contribute to making the abstract and the inexplicable more concrete and comprehensible. The subthemes also include codes highlighting patients’ experiences of factors that make satisfactory interaction between patients and healthcare professionals impossible, causing the patient to feel uncertainty. Together, these subthemes illustrate the wide range of variation in patients’ experience of ward rounds.

**Figure 2:**
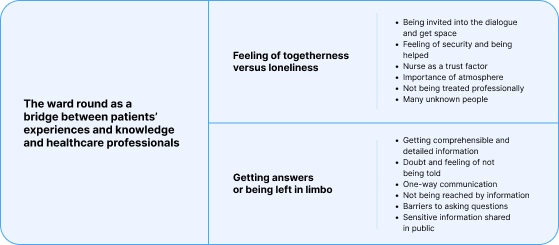
Illustration of themes including codes.

The subthemes presented included the codes generated for the respective subthemes (figure 2).

### The ward round as a bridge

Patients’ experiences of ward rounds were described as multifaceted, ranging between feeling included and being left out. A bridge can be used as a metaphor for this. A bridge can enable meetings between healthcare professionals and patients in which both parties are involved. A bridge can also be fragmented, with a narrow passage that cannot accommodate both the professionals and the patients, meaning that one party is forced to wait their turn or ends up lagging behind. If there is no bridge, the healthcare professionals and the patient cannot meet and there will be no exchange of knowledge.

### Feeling of togetherness versus loneliness

The round was experienced as a positive occurrence during the hospital stay and an asset to being cured and becoming healthy. Patients felt encouraged and strengthened by the rounding healthcare professionals. Being invited into the dialogue and get space to share their experiences was highlighted as important by several patients. In this regard, patients emphasised their own responsibility in being open, prepared and relating their experiences. Therefore, patients’ personal characteristics were also important for being able to speak up.

Don’t keep it bottled up inside… so if you have a question or think about something, it’s incredibly important to say it. […] if there is any concern or if you wonder about something, something that isn’t clear, it is very much on your own…own responsibility there too, and if you don’t ask, you won’t find anything out, to use a cliché, but… (Patient 3)

Being asked about how one experienced one’s own well-being was appreciated. Patients experienced that it was important to discuss different perspectives on their health, not just the medical one, and seeing the whole picture:

[…] Perhaps you should think a little outside the box, what the full picture for the patient is, […] when you get home, what you should do to take care of your health and move more and to eat healthier and things like that, if they included that in the picture as well, so that you can prevent things like this from happening again. (Patient 11)

The informants stated that the content and atmosphere of ward rounds did not depend only on the patient’s personality, but also on the personality of the doctor and the nurse. Personal chemistry was described as important for the patient’s sense of being invited into the dialogue and being given space.

From the patients’ point of view, the doctor was the professional who had knowledge and the one who related the most important details about treatment and complications. Seeing a doctor was important to the patients and the doctor’s expertise could contribute to a feeling of security and being helped. The informants said that they felt that the doctor invested in time on rounds and did not come across as stressed. If a doctor sat down instead of standing by the edge of the bed, that had a positive effect.

He took a chair and sat next to my bed. Just so there was some peace and quiet and that felt great. And then I felt what a difference it was to talk to him compared with the one standing by the door. (Patient 2)

Even though ward rounds were strongly associated with seeing a doctor, the nurse’s presence at the round felt valuable as a trust factor. The patients perceived the nurse as their representative and a connection to the doctor that was important for their health development. The nurse was often perceived as quiet and withdrawn, but some patients believed that the nurse should be given more space on the rounds and saw the nurse’s function as very important.

Very important, much more important than the doctor is, because they…they know their job, they usually see how we feel, at a detailed level, I mean they can go in and see things, the doctor only sees us five or ten minutes per day, but the nurses see us all the time and they are…very good and competent, so the doctors should listen more to the nurses. (Patient 10)

The informants said that it is not only the contents of the round that were important, but also how it was carried out and the general atmosphere. Staff running in and out of the room was perceived as annoying and meant that what came up during rounds did not reach all the healthcare professionals, who would then be forced to ask others. To avoid this, patients wanted everyone concerned to participate in the rounds and get the same information. The patients felt that the rounds were important for daily updates and looked forward to them.

And we discussed it in the room where I was, because one day the round didn’t come and it felt very strange, because everyone wondered why it didn’t come and what had happened, why we didn’t get a ward round this morning, or this afternoon, for that matter. (Patient 7)

The physical place where the round was carried out was not of great importance to most patients. Some patients felt that it was nice and safe to lie in their bed and wanted ward rounds to take place there rather than in another room. On the other hand, some patients said that the experience of being in bed with staff standing around caused a feeling of inferiority and awkwardness. It was said that being surrounded by several people with serious facial expressions could cause fear of receiving a death notice, making communication difficult. Several patients asked for less serious facial expressions and involving elements of humour to create a good atmosphere.

Because if you do, the dimples appear and you’ll laugh and you’ll remember it just because of that. (Patient 15)

To avoid destroying this good atmosphere, some patients did not dare criticise the ward rounds at the hospital where they were now, but they could do this when talking about their earlier experiences in other hospitals. Also, if they did not like something at the current hospital, they excused the staff:

I can’t…complain about them […] And that they do everything they can, all the staff do that […] Yes, sometimes they can…well, I don’t know if they feel rushed, but I understand if they do sometimes, it’s a lot, they have a lot to do. (Patient 5)

The patients stated that they felt respected and involved, with the experience of being confirmed and listened to being significant. The patients also experienced a sense of not being treated professionally. Nonchalant and distant behaviour and the feeling that the doctor was not up to date were described as creating an uncomfortable sense of uncertainty. Likewise, the importance of choosing one’s words with care was highlighted.

…The doctor was standing, leaning against the bathroom door there and then he told me ‘you should know that you have a cancerous tumour’, he said. It wasn’t a good way to say that…you are usually called into a room and get to talk it through in peace and quiet, as it were. So that made me a little sad, actually. (Patient 2)

The patients’ experiences of and opportunities for dialogue were affected by which actors participated and, above all, how many they were. The interviews revealed that many people participated in ward rounds, and they did not always introduce themselves. Some patients described it as the doctor leading the way with an entourage in tow. To understand who the unknown round participants were, the patients tried to read name tags. Unknown participants in the rounds could distract the patients and the absence of the ‘right’ participants could pose a risk that important information would not be presented.

No, but I thought that, what are they here for, these people I’ve never met. I thought some of my nurses should have been there instead. Because they could have added something that I had forgotten. (Patient 14)

Some patients said that they wished there were fewer participants during rounds and highlighted the doctor, nurse and assistant nurse as important participants. The patients understood that some participated for educational purposes but wished to be asked permission when this occurred.

Then you should ask, I think, if you have others who want to come to get…well, if it’s education, or whatever it is, using a patient, then you should always ask the patient if it’s okay, but you don’t do that, not here. (Patient 9)

### Getting answers or being left in limbo

Several of the patients said that the doctor expressed detailed information that was also comprehensible. Positive experiences from rounds were associated with receiving positive news regarding the disease—finding out the test results look better, getting clarity on which action is relevant, and information about when it could be time for discharge. The patients also emphasised the importance of the doctors being well-prepared, expressing themselves correctly and choosing their words carefully.

That the doctor talks about how the situation is and does it in a good way, so it doesn’t make you sad. I think that’s important. Then today another sweet doctor came up to me and he, he was so nice and caring in a way and I actually appreciated that. And he didn’t talk about cancerous tumours here and there, just like that, but he said the ‘change’ that’s in your lung… and that’s what it is, right, since we still don’t know what it is. (Patient 2)

Patients experienced that it could be difficult to absorb, understand and remember information given during rounds. Even if the patient understood the information, it could be difficult to sort it all out on their own, especially when it came to making decisions. Patients highlighted the value of having relatives with them during rounds, but not knowing the exact time when the rounds would take place, and geographical distances made it difficult for relatives to participate.

[…] So sometimes it’s good to have a relative with you, because there can be a lot of information that you get and there are things you have to make a decision on, which may not be easy to sort out yourself. […] absolutely, the support of relatives may sometimes be needed. (Patient 4)

The experience of receiving good, comprehensible, continuous information from the nurses on the ward contributed to some patients saying that it was not necessary to have rounds every day. If nothing much was happening with the disease or treatment aspects, such as adjustment of medication, patients felt that the frequency of rounds could be reduced to every 2 days or maybe even every 3 days. Knowing which treatments or interventions were planned as well as getting information on when something might take place was important, to be able to plan and prepare.

Yes, so what I think is particularly good is that I get so much information. And I like that. Because I want to know what is happening to me. And I really get to know that. (Patient 6)

Although detailed information was appreciated, the patients understood that some questions could not be answered. At the same time, doubt and a feeling of not being told without asking were described. Patients had a sense that healthcare professionals received more information than they themselves did during rounds. The patients wanted more complete information and described the ward rounds as brief. It also emerged that even if patients felt they were being informed, doubts could arise as to whether the information was correct.

Yes, I would say that I get the information I need. But then you don’t know if they are holding back on the truth or what they know. One can only hope that they say what they believe and think. (Patient 1)

It was explored that the rounds could be experienced as an occasion when the doctors gave information, and the patients saw themselves as recipients who could only contribute by asking questions—if they had any. The feeling of not really being involved in the dialogue and being forced to interrupt and say ‘now I have to talk, so that you understand me’ indicates an imbalance in the interaction. The patients said that they felt left out and had to accept that. Some patients said that they were not heard, describing a one-way communication and being forced to get angry in order to be listened to.

Many patients, like me, who have been in and out of hospital due to disease for many years…I know my body, and many doctors don't listen until you get really angry with them and then they can listen, and some of them are very nonchalant. (Patient 10)

The findings showed that information given at rounds did not always reach the patient. The information could also be difficult to understand because it was given in a way that the patient did not understand. Not understanding whether a ‘major operation’ was major to its extent or its risk not only raised many thoughts but could also create anxiety. Lack of awareness about a patient’s previous care experiences could lead to information not reaching the patient.

[…] Then they talk a little about how… (thinking) yeah, how things are going with samples and stuff like this. But I’m the one, so I don’t know what they’re talking about really, huh, what tests? I’ve never really been sick. (Patient 1)

The findings showed that, although patients wanted to know more, they experienced barriers to asking question*s*. Impaired hearing and difficulty understanding what the staff said, as well as an impaired general condition, were reasons for this. Others had a fear of causing offence, not being properly understood or having to apologise.

No, then the thing is that…and I have it…it clear to me, but…and I understand that there are things they are investigating and so on, so it’s a little…it’s a hierarchy, it’s not just anyone who can say something, right? It could perhaps be that the hierarchy is such that you can say something, because then you have to wait, now he’ll be here tomorrow at two o'clock instead. (Patient 13)

Whether rounds took place in private or on a ward where other patients could hear what was said was important to some patients. If rounds took place in a room with other patients, that meant the patient was forced to share sensitive information with copatients. This was perceived negatively and as something that could create discomfort. The presence of other patients also affected what the patient would choose to bring up and tell and which questions they would ask. The patients accepted this, but wished it was different.

[…] So you hear what they’re saying about your fellow patients too, and that doesn’t feel great, because I’m not supposed to know why that person is hospitalized, and they shouldn’t really know why I’m there, if I don’t choose to tell them, but it’s hard to get away with the way it is, because then you’d have to have the rounds in some other way, or ask that person to leave the room… (Patient 4)

## Discussion

The aim of the present study was to explore patients’ experiences of ward rounds in inpatient care. The study findings revealed that rounds clearly filled an important function for patients. Patients experienced rounds as a bridge between themselves, their knowledge and the professionals. Similar results were shown in the Australian study indicating that patients value ward rounds as the opportunity to interact with healthcare professionals.^7^ The findings also indicated that the patients were primarily recipients of information, which is congruent with earlier research in Sweden by Swenne and Skytt^2^ and differs from results of the Australian study^7^ which shows that more experienced patients in using healthcare systems are more engaged in care planning and asking questions. Swenne and Skytt^2^ highlighted the need to change the ward round routines to allow more time for a two-way process of information exchange.

However, in the present study, it appeared that the patients felt that there was enough time during rounds. To our knowledge, this is a finding not shown in earlier research. This indicates that patients’ opportunities to participate in dialogue may depend on other factors. Such factors can be trust in healthcare professionals and patient satisfaction with their care. These factors can even lead to lower patients’ participation. According to the study by Becker *et al*,^10^ most patients want to participate in decision-making, but—paradoxically—a patient’s degree of participation is higher if their trust and satisfaction with healthcare professionals are lower; it does not depend on their knowledge of medical care. In line with that, the present study showed that patients were satisfied with the rounds when they perceived a permissive atmosphere and got support from healthcare personnel as well as detailed and comprehensible information about their own health. This can explain several paradoxes shown in the findings, which, to our knowledge, have not been shown in other research. First, patients feel disadvantaged when they cannot participate in decision-making, unless they feel such trust for healthcare professionals that they can hand over decision-making to them. Second, patients do not dare to criticise professionals even if they do not receive all the necessary information, as they are afraid of ruining their relationships with the professionals.

In the present study, participation was also associated with being able to contribute information, being able to answer questions and feeling listened to. Receiving information thus seemed to be sufficient for some patients, whereas others wanted to be activated and be able to make contributions themselves. Every patient is unique, and care should, therefore, be adapted to suit each patient’s needs. Even patients with the same diagnosis may need to receive different care due to their individual biological, psychological and social prerequisites.^23^ This is congruent with patient-centred care—the components of which are patient involvement and individualised care.^11^ If this is applied to rounds, they can become patient-centred in that patients’ needs and wishes are taken into account. Clinicians should consider the factors that could make rounds more patient-centred and apply them in practice.

Furthermore, the findings of this study show that the ward rounds did not always meet the patients’ expectations and that this could create a feeling of being left in uncertainty instead of contributing to a sense of increased control over their situation. This caused patients to feel that they were left in limbo instead of getting answers. This finding is supported by a study from Australia, which showed that patients sometimes did not understand what the professionals were saying and did not have an opportunity to ask questions during rounds.^7^ Also, similar findings were made in a Swedish context in a qualitative study where students were interviewed, as well as in studies investigating patients’ experiences and perceptions of the ward rounds and their possibilities to participate therein.^2 24 25^ A study by Hordiienko *et al*^24^ showed that students perceived patients as not fully involved in rounds due to the difficult medical language and not understanding the routines. Swenne and Skytt^2^ showed that the information given to patients during rounds was not sufficient and that they needed to meet nurses and doctors again, to get supplementary information. According to Aronsson *et al*,^25^ not all patients can participate in rounds and there is a need for better communication and an individual approach to patients. Further studies should be conducted to understand the factors that make information comprehensible for patients during ward rounds and facilitate patient participation in rounds, so these factors can be implemented in the round routines.

One factor that contributes to patients’ non-participation in the ward rounds is already known to be the complicated medical content.^2 24^ In the present study, rounds were described by patients as being focused on medical aspects and an occasion when a doctor informs about diagnosis and disease development, results of examinations and sampling, planned measures and treatment, and prognosis. This is also what the patients associated with the term ward rounds. There was thus an obvious power imbalance due to the one-way communication and the medical focus, meaning that it was not unexpected that patients sometimes felt left out. The experience of not being invited into the conversation, not getting clear and comprehensible information and not being able to ask questions contributed to this and could create a feeling of not being included. This is in breach of Swedish law, which states that care should be based on the patient’s co-determination and meet the patient’s needs.^26 27^ According to the present study, when rounds were conducted without patients being invited into the dialogue, it caused patients to feel loneliness instead of togetherness. Similar findings have been indicated in previous studies, showing that patients feel excluded from the discussions that take place during rounds^3 6^ and do not feel seen as individuals.^6^ Other studies confirm that use of specialist language, a lack of information transfer, limited opportunities to ask questions and a focus on the illness instead of the individual are perceived as obstacles to patient participation.^3 4 6 28^

### Study strengths and limitations

To our knowledge, there are few studies in the Swedish context that explore patients’ experiences of ward rounds.^2 20^ Our study can contribute to the knowledge about patients’ experiences of ward rounds and increase understanding of what is important for patients during rounds.

The discussion regarding the study quality is inspired by Lincoln and Guba^29^ and the discussion regarding the analysis is inspired by Braun and Clarke.^21 30^

The sampling in the study was purposeful,^22^ meaning that informants who had experiences of ward rounds and were able to answer the research question were recruited. Therefore, it was necessary to involve gatekeepers to identify patients, meaning that selection of patients to some extent rested on external third parties. This may affect credibility due to preunderstanding of the gatekeepers. The patients considered not suitable by the gatekeepers were not asked to participate and, therefore, their experiences may not be shown in the findings. Still, as the gatekeepers were patient care coordinating nurses who knew patients well, it contributed to patients’ feeling comfortable to ask questions or to decline participation in the study as well as to an increased possibility of reaching the desired population and capturing variations in age, ward experience and gender. Further, it increases credibility, thanks to the prolonged engagement of the gatekeepers and their understanding of the culture and settings.

To participate in an interview, the patients needed to be verbally and cognitively capable and speak Swedish, which means that patients who could not take equally active roles in ward rounds due to, for example, language difficulties, cognitive function or foreign origin did not have the opportunity to share their experiences. Further, participants were recruited from the single hospital, though from different departments: medical and surgical, to get the variation in the sample. The results can thus be seen as transferable to similar populations and contexts but might not apply to those not represented in the study. Transferability and confirmability were promoted through thick descriptions of the study population and context as well as data collection and analysis.

Dependability was promoted by the authors’ reflections and discussions during all stages of the study process: study planning, recruitment, data collection, analysis and reporting. The two first authors wrote reflexive journals. This promoted the authors’ reflexivity and revealed their preunderstandings.

Recording of interviews means that the interviewer could focus more on non-verbal communication and thus catch things that might have been missed if notes were taken in parallel, such as emotional outbursts, pauses and verbal nuances.^29^ Likewise, the recordings contributed to fidelity by making it possible for the researchers to listen afterwards, which is important when analysing data. This increases trustworthiness. Recorded data also make it possible to use quotes to confirm the result, increasing confirmability.^29^

Each interview’s length was regulated by the patient. They could be considered short, which could influence the analysis due to the potential lack of data. However, the contents of the interviews were sufficient for the exploration of the patients’ experiences of ward rounds.

Research based on thematic analysis (TA) is always shaped by the researcher; the researcher is never completely neutral due to always being influenced by their subjectivity. Therefore, subjectivity is considered an important factor for successful reflexive TA.^21 30^ The first two authors engaged in the exercise ‘critical friends’, to ensure reflexive TA quality. This is a dialogue in which researchers reflect on their interpretations and perceptions of the data, give feedback to each other and challenge each other’s interpretations.^31^ To clarify the authors’ perspective, the analysis was located theoretically in critical realism, as described in the experiential qualitative approach.^21^ As critical realism presupposes that reality is accessible through the different perceptions and experiences that people have about it, all the authors continuously discussed their preunderstandings and reflected on the analysis to identify the values and assumptions that were taken for granted.^21^

It was described in the analysis of the code importance of atmosphere that some patients did not dare to criticise rounds at the hospital where they were admitted at the time, and even if they did not like something, they excused the professionals. This part of the analysis required more latent coding of meaning than the other parts. However, according to Braun and Clarke,^21^ coding should not be considered either semantic or latent—it can be both. This could have influenced the results of the study by showing more positive patients’ experiences. Still, there were patients who could criticise ward rounds. This reluctance to critique could reflect the behaviour and inclinations of patients in general and points out that more attention should be paid to it by healthcare professionals at ward rounds, as well as communication should be adapted accordingly. The fact that the interviews took place in close connection with rounds increased the possibility of capturing the patients’ experiences and reduced the risk of impressions and feelings being forgotten.

## Conclusions

The findings of the present study show that from patients’ perspective, ward rounds in inpatient care play an important role for patients’ care and health, but patients’ experience can be varied and contradictory: feelings of togetherness versus loneliness and getting answers versus being left in limbo. The findings show that it is important to develop a model of rounds in clinical settings which can contribute to an open and supportive atmosphere, when patients are invited into the dialogue, treated professionally and friendly, supported, given time and attention, and their feelings are respected. Patients experienced that sharing of comprehensible and detailed information in full as well as having the opportunity to ask questions is important for them at ward rounds.

Further studies could focus on the healthcare professionals’ experiences and perspectives of ward rounds in inpatient care as well as patient participation therein.

## Supplementary material

10.1136/bmjopen-2025-103481 online supplemental file 1

## Data Availability

No data are available.
