# Supplementary material for: Patients’ experiences of ward rounds in a Swedish context: a qualitative study
Source: BMJ Open. 2025 Nov 13;15(11):e103481. doi: 10.1136/bmjopen-2025-103481 (PMC12625863; doi:10.1136/bmjopen-2025-103481)
Supplement: online supplemental file 1 [file bmjopen-15-11-s001.docx]

**Supplement 1**

**Patients’ experiences of ward rounds in a Swedish context: A qualitative study**

**Interview guide patients**

Healthcare department___________________________ Age: _______________

Reason for hospitalization ______________________________________________

What do you think when you hear the word “round”?
*- Who participate in the round?
- What are your expectations for the round?*

Could you tell me about how rounds are performed here at the ward?
*- What feelings do you get when the round enters the room, and the focus is on you?
- What is said during the round?*

How do you see your own role and participation in rounds? *- Do you bring up anything during the round and if so, what?
- Is there any information you are missing during the round?*

Can you tell me about a positive round situation? / Is there any round that you have experienced particularly well? *- What did you find particularly good?
- How did it feel for you?*

Can you tell me about a negative round situation? Is there a round that you have experienced as particularly bad?
*- What did you find particularly bad?
- How did it feel for you?*

Is it important for you to have rounds every day?
 *Why do you think so?*

How would you like rounds to be performed?
*- In the wardroom? In a separate room where patients are allowed to enter one by one?
- What do you think is important to address during rounds?*
