# Supplementary material for: Patients’ experiences of ward rounds in a Swedish context: a qualitative study
Source: BMJ Open. 2025 Nov 13;15(11):e103481. doi: 10.1136/bmjopen-2025-103481 (PMC12625863; doi:10.1136/bmjopen-2025-103481)
Supplement: Supplementary file 2 [file bmjopen-2025-103481.reviewer_comments.pdf]

PEER REVIEW HISTORY

BMJ Open publishes all reviews undertaken for accepted manuscripts. Reviewers are asked to complete a checklist review form and are provided with free text boxes to elaborate on their assessment. These free text comments are reproduced below.

ARTICLE DETAILS

Title (Provisional)

Patients’ experiences of ward rounds in a Swedish context: A qualitative study

Authors

Larsson, Emma; Hordiienko, Yelyzaveta; Fagerström, Cecilia; Almerud-Österberg, Sofia; Finnbogadóttir, Hafrún Rafnar; Persson, Carina

---

VERSION 1 - REVIEW

**Reviewer**	**1**
**Name**	**Vangompel , Emily White**
**Affiliation**	**University of Illinois Chicago**
**Date**	**19-Jun-2025**
**COI**	**None**

Overall, this manuscript studies a topic of interest in a new context. It is particularly strong in its discussion of how it incorporated reflexivity into the thematic analysis. The chosen quotes helped to illustrate and support the authors’ analysis. Below I detail a few areas that need additional detail, but I think much of the discussion could be shortened as there are many different places where the same findings and interpretations are repeated.

Abstract:

The abstract lists 4 strengths of the study but no limitations. Consider adding limitations for completeness.

Introduction:

The description of different types of ward rounds seems incomplete. How does this relate back to patients’ experiences of rounds? Are there studies that demonstrate different types or structures of rounds result in different patient experiences?

Line 43 – “Interactions with the healthcare professionals…” Can you explain the dichotomies presented in this sentence, particularly the importance of “having knowledge” versus “being informed”?

Pg 4, Line 26 - The case for how this exploratory analysis will improve quality and safety is only briefly mentioned and not supported with existing studies.

Aim: The introduction contains a lot of background that doesn't seem to tie back to the Aim. I'm not sure how the previous discussion of different types of rounds and different types of patient participation preferences in care directly justifies the study aim, which seems like it has already been explored. If the aim was to study this issue in a European context, perhaps the background should include more about why the European context may have unique contextual components that impact patient experiences of rounds.

Design: Do you mean "exploratory descriptive" instead of "explorative"?

Participants/sampling:

- For the "stratified purposeful sampling" please describe your stratification and sampling process.
- Who could act as "a gatekeeper" in determining who could be approached to participate?

Data Collection and Settings:

Can you clarify if part of the data collection involved study team members observing or attending rounds? If so, how may this have affected the performance of rounds? If not, how was the information regarding the structure of the rounds obtained?

How was the preliminary interview guide developed? Was an existing theory or framework used? Were decision control preferences, as described in the introduction, incorporated into the interview guide development?

Data Analysis:

I found the description of the data analysis to be strong. Several different theories and approaches that informed data analysis are named and referenced; however, the resulting analysis is relatively straightforward. It would be nice to see a figure (perhaps adapting Fig 1) to include how and at which points the different approaches and reflexivity were incorporated into the analysis.

Results:

I would avoid use of "obviously" in regard to stating results.

It came as a surprise that patients were describing previous experiences at other hospitals in the results section, as it seemed that

Discussion:

Pg 15, last paragraph, first sentence, needs a reference.

Pg 16, second paragraph: I find the use of the word “warm atmosphere” to be vague. Can you replace it with the specific aspects or contents that create the atmosphere conducive to patient participation and/or satisfaction?

Pg 16, “The sampling in the study was stratified purposeful…” the rest of the sentence does not describe stratified purposeful (sometimes called stratified purposive) sampling, but rather a convenience sample. As noted above, the stratification scheme and sampling method needs to be spelled out with greater detail. How many and what were the strata? How many individuals were sampled from each strata? From there, were any adjustments made to ensure representation from each strata?

Pg 17, first paragraph discussing the use of gatekeepers to identify patients: In methods, please describe gatekeepers with greater detail. In this limitations section, please describe how this could bias your results so that the reader can understand both the potential bias and why it was justified and make their own decisions regarding credibility.

Pg 18, second paragraph: this fear of criticizing the hospital in which they were currently admitted is a significant limitation introduced by the decision to hold interviews while the patient was still admitted. Please include a discussion of how this could have biased results in this section. I understand that you have included it as part of your analysis, but it should also be detailed as a significant limitation.

Conclusions:

Pg 18, third paragraph: “The findings show that it is important to develop rounds in clinical settings,” is vague. I’m not sure in what way you are recommending rounds to be developed.

Figures:

Figure 2 seems redundant with Table 1.

**Reviewer**	**2**
**Name**	**Nanayakkara, Prabath**
**Affiliation**	**Amsterdam UMC Locatie VUmc**
**Date**	**26-Jun-2025**
**COI**	**None**

Well written manuscript, clearly articulating the importance of ward rounds for patient care and patient-centredness. The research question is very relevant and timely, addressing a gap in the international and Swedish context. The use of the explorative descriptive design with semi-structured interviews and reflexive thematic analysis is appropriate for the aim of

investigating this theme. The study provides detailed patient quotes and nuanced insights into the diversity of patient experiences. I quite like some statemnets such as "He took a chair and sat next to my bed. Just so there was some peace and quiet and that felt great. And then I felt what a difference it was to talk to him

compared with the one standing by the door" which was a simple intervention with a profound effect on the perception ofthe patient. There are a few weak point such as sample size is small, homogenous (swedisch speaking) from a single hospital limiting external applicability. Gate keeper selelction and patient`s relucatnce to critisize may skew the findings towards more positive expeirience. Why weren`t health care professional interviewed and were observes used to observe the discusions (Or are you planning to publish these later?). In conclusion, The study is a scientifically robust and societally relevant exploration of patients' experiences of ward rounds in Swedish inpatient care. I would like to commend authors for this relevant piece of work.

**Reviewer**	**3**
**Name**	**PAUTRAT, Maxime**
**Affiliation**	**Université de Tours Faculté de Médecine**
**Date**	**13-Aug-2025**
**COI**	**None**

Thank you for giving me the opportunity to discover this study.

This topic is relevant for improving the shared decision-making strategy between patients and medical staff during hospitalization. However, experiences can vary greatly depending on whether the medical team adopts a multidisciplinary approach, with or without the presence of numerous students. These considerations limit the data to medium-sized hospitals, as described in this study.

Method: the interview guide seems appropriate. The authors appear to be well -trained in qualitative methods. Why did 20 patients decline to participate? Medical and surgical context: patients' experiences can vary considerably depending on the type of surgery or disease... It may be necessary to describe these contexts in more detail. The patients' experiences could have been explored much more effectively using a GTA approach rather than a thematic analysis.

Results: easy to read, but too many comments from the authors, which should be shortened. More space should be given to verbatim quotes.

Very short interviews (median 15')... and only 16...

Thematic analysis: the least in-depth of the analytical prisms... (why not a GTA???)

So the range of results is limited : fig 2...

The limitations were very quickly listed in the discussion section...

The perspectives were also weak: continue to develop "an open and supportive atmosphere and sharing of comprehensible and detailed information"

= Critical limits for the editorial board?

VERSION 1 - AUTHOR RESPONSE

**Reviewer 1**			
**#1** Overall, this manuscript studies a topic of interest in a new context. It is particularly strong in its discussion of how it incorporated reflexivity into the thematic analysis. The chosen quotes helped to illustrate and support the authors' analysis. Below I detail a few areas that need additional detail, but I think much of the discussion could be shortened as there are many different places where the same findings and interpretations are repeated.	Thank you for yor valuable feedback on the manuscript. We have shorten some part of Discussion.	Page 17<br><br>Page 16<br>Lines 9-17	**The last paragraph of the discussion was erased.**<br><br>**The following two sentences were transferred from the last to the second paragraph of Discussion:** In line with that, the present study showed that patients were satisfied with the rounds when they perceived a permissive atmosphere and got support from healthcare personnel as well as detailed and comprehensible information about their own health. This can explain several paradoxes shown in the findings, which, to our knowledge, have not been shown in other research. First, patients feel disadvantaged when they cannot participate in decision-making, unless they feel such trust for healthcare professionals that they can hand over decision-making to them. Second, patients do not dare criticize professionals even if they do not receive all the necessary information, as they are afraid of ruining their relationships with the professionals.
**#2 Abstract:**<br>The abstract lists 4 strengths of the study but no limitations. Consider adding limitations<br>for completeness.	We agree it was incomplete. We have added the limitations.	Page 1- 2<br>Lines 34-37<br>Lines 3-6	**Please, see the answer to the Editor(s) comment #1.**
**#3 Introduction:**<br>The description of different types of ward rounds seems incomplete. How does this	We agree this is incomplete. We have explained that only some kinds of rounds include patients' involvement, which may	Page 3<br>Lines 13-16	**The following text is added:**<br>Only the ward rounds with a multidisciplinary team involve patients in treatment and discharge plans discussions while other types of rounds, for example, consultant and teaching rounds focus on communication between

relate back to patients' experiences of rounds? Are there studies that demonstrate different types or structures of rounds result in different patient experiences?	influence patients' experiences.<br><br>We have also clarified to what type of rounds refere patients' experiences mentioned in the text.	Page 3<br>Lines 24-26	healthcare professional who discuss patient care, which may influence patients' experiences [5].<br><br>These experiences concern multidisciplinary rounds, where several professions participate, which are in focus of the present study.
**#4** Line 43 – "Interactions with the healthcare professionals…" Can you explain the dichotomies presented in this sentence, particularly the importance of "having knowledge" versus "being informed"?	We have explained the dichotomies which can make the text clearer.	Page 3-4<br>Lines 30-34<br>Line 1	**We have revised as following:**<br>**Before:** Interactions with the healthcare professionals are often emphasized rather than participation in decision-making, and having knowledge is emphasized rather than being informed [9].<br>**Now:**<br>Interactions with the healthcare professionals, meaning sharing the same care aims, respecting each other for knowledge and mutual contribution to communication, are often emphasized rather than participation in decision-making [9]. Having knowledge is emphasized rather than being informed, meaning that comprehending information and knowing where to turn for help and what to do to get well is more important for patients than receiving personally tailored information [9].
**#5** Pg 4, Line 26 - The case for how this exploratory analysis will improve quality and safety is only briefly mentioned and not supported with existing studies.	We have explained why icreased knowledge of patients' experiences of ward rounds can contribute to care quality and safety and supported it with literature.	Page 4<br>Line 28-30	**Following sentence is added to the introduction:**<br><br>Furthermore, when patients are engaged in their care it contributes to their health outcomes, positive experiences of care and safety [17, 18].
**#6 Aim**<br>The introduction contains a lot of background that doesn't seem to tie back to the Aim. I'm not sure how the previous discussion of different types of rounds and different types of patient participation	Yes, there were studies mostly outside Europe, therefore studies in European context are needed, which was even highlighted by the systematic review by Heip et al. (2022).	Page 3<br>Line 22<br><br>Page 4	**We have mentioned an Australian study:**<br><br>It also appears according to the Australian study that patients feel uncertain about their role during rounds and that their experiences of ward rounds are important for their commitment, expectations, and opportunity to participate [7].<br><br>**The following text was added to Introduction:**

preferences in care directly justifies the study aim, which seems like it has already been explored. If the aim was to study this issue in a European context, perhaps the background should include more about why the European context may have unique contextual components that impact patient experiences of rounds.	We have clarified that in Introduction an Australian study is mentioned.<br>We have added the clarification why the European context may have unique components that may influence patients' experiences of ward rounds.<br><br>Further, in Discussion we have compared findings of the Australian study with our findings.	Lines 23-27<br><br>Page 15 Lines 29-30<br><br>Page 15 Lines 30-34	Healthcare system in Europe has strong primary care and universal coverage, while, for example, Australian healthcare system relies both on public and private hospitals [15, 16]. The mentioned aspects of the European healthcare system may have impact on the ward round organization and, therefore, on patients' experiences of the rounds.<br><br>**We have added the following text:** Similar results were shown in the Australian study indicating that patients value ward rounds as the opportunity to interact with healthcare professionals [7].<br><br>**And revised as following:**<br>**Before:** The findings also indicated that the patients were primarily recipients of information, which is congruent with earlier research in Sweden by Swenne and Skytt [7] …<br><br>**Now**: The findings also indicated that the patients were primarily recipients of information, which is congruent with earlier research in Sweden by Swenne and Skytt [7] and differs from results of the Australian study [7] which shows that more experienced patients in using healthcare system are more engaged in care planning and asking questions.
**#7 Design**<br>Do you mean "exploratory descriptive" instead of "explorative"?	Yes, we meant exploratory descriptive design (Hunter et al., 2019).	Page 1 Line11<br><br>Page 5 Line 4	**Design.** An exploratory descriptive design was chosen.<br><br>An exploratory descriptive design was chosen to explore patients' experiences of ward rounds using semi-structured interviews [19, 20] and reflexive thematic analysis [21].
**#8 Participants/sampling:** For the "stratified purposeful sampling" please describe your stratification and sampling process.	Thank you for drawing our attention to this! We have revised and the sampling of the study was purposeful and captured information-rich cases to explore patients' experiences. Patients were selected with variation in age, gender and healthcare experience.	Page 5 Lines 9-10	**We have revised as following:**<br><br>**Before:** In the selection of patients, as wide a spread as possible was sought regarding age, gender, and healthcare experience. A stratified purposeful sampling was therefore used, as it is useful for capturing variations rather than similarities [22].<br><br>**After:** A purposeful sampling was used to capture information-rich cases for exploration patients' experiences of ward rounds [22]. In the selection of patients, as wide a spread as possible was sought

			regarding age, gender, and healthcare experience.
**#9** Who could act as "a gatekeeper" in determining who could be approached to participate?	We have described the gatekeepers in greater details in Participants/Sampling	Page 5 Lines 20-21	**We have added the following sentence to Participants/Sampling:**<br><br>The gatekeepers were patient care coordinating nurses, who have the continuous and overall responsibility of a separate patient care, its planning, conducting, assessment and evaluation.
**#10 Data Collection and Settings:**<br><br>Can you clarify if part of the data collection involved study team members observing or attending rounds? If so, how may this have affected the performance of rounds? If not, how was the information regarding the structure of the rounds obtained?	Though the interviews were conducted by two research nurses who did not work at the selected wards and had no relations to the participants, still they had knowledge of how the rounds are conducted from the communication with colleagues and gatekeepers. Furthermore, they got information about the structure of the rounds from patients during interviews. Observations have not been performed during this study and are planned to be conducted in further studies of ward rounds.	Page 6 Lines 23-27	**We have revised as following:**<br><br>**Before:** The interviews were conducted by two research nurses separately (each conducted eight interviews) who did not work at the selected wards and had no relations to the participants.<br><br>**After:** The interviews were conducted by two research nurses separately (each conducted eight interviews) who did not work at the selected wards and had no relations to the participants, still they had knowledge of how the rounds are conducted from the communication with colleagues and gatekeepers. Furthermore, they got information about the structure of the rounds from patients during interviews.
**#11** How was the preliminary interview guide developed? Was an existing theory or framework used? Were decision control preferences, as described in the introduction, incorporated into the interview guide development?	The preliminary interview guide was developed by focusing on categories of patient-centred care in patients' definitions given in Robinson et al. (2008). Questions about the decision control preferences were not incorporated in the interview guide directly, still they could be understood and captured from the answers patients gave at the interviews.	Page 6 Lines 10-13.	**We have added the following text to the Data collection and settings:**<br><br>While developing interview guide the researchers were focusing on categories of patient-centred care defined by patients, such as involvement in treatment, information provided, partnership, respectful treatment, communication, patient as priority, accessible care [11].<br>With these questions patients' decision control preferences could be captured, though no direct questions were created aiming to this.<br><br>**As well as have given one more example of the question from the interview guide, which was aimed**

			**to receive information about communication, information provided, patient as priority, etc.:** *Can you tell us about a positive round situation? / Is there any round that you have experienced particularly well?*
**#12 Data Analysis:** I found the description of the data analysis to be strong. Several different theories and approaches that informed data analysis are named and referenced; however, the resulting analysis is relatively straightforward. It would be nice to see a figure (perhaps adapting Fig 1) to include how and at which points the different approaches and reflexivity were incorporated into the analysis..	Thank you for the positive feedback. Figure 1 was modified to include writing reflexive journals, the exercise ”critical friends”, reviewing themes in comparison with codes, the research question and full data set as well as critical dialogue with all the research group.Experiential qualitative theoretical approach as well as critical realism were shown in Figure 1 as they were informing the analysis.	**Please see Figure 1.**	
**#13 Results:** I would avoid use of “obviously” in regard to stating results.	Thank you for this remark. We have deleted “obviously	Page 12 Lines 19-20<br><br>Page 13 Lines 26-27<br><br>Page 14 Lines 18-19	**We deleted “obviously” in the sentence:** The patients’ experiences of and opportunities for dialogue were affected by which actors participated and, above all, how many they were.<br><br>**Wrote ”Patients experienced” instead of ”It was obvious” in the sentence:** Patients experienced that it could be difficult to absorb, understand, and remember information given during rounds.<br><br>**We have sabstituted ”It became obvious” with ”it was explored”:** It was explored that the rounds could be experienced as an occasion when the doctors gave information, and the patients saw themselves as recipients who could only contribute by asking questions – if they had any.
**#14** It came as a surprise that patients were describing	Yes, it was unexpected, but it shows that patients were free to talk and describe	Page 18 Lines 20-22	**We mantioned about different departments to get the vatiation**

previous experiences at other hospitals in the results section, as it seemed that.	their experience of ward rounds. With the focus in data collection and analysis being on experiences of rounds related to ongoing care, patients' spontaneous reflections on previous experiences contributed to clarifying the current experiences.		**in the sample in Study Strengths and Limitations in Discussion:**<br><br>Further, participants were recruited from the single hospital, though from different departments: medical and surgical, to get the variation in the sample.
**#15 Discussion:**<br>Pg 15, last paragraph, first sentence, needs a reference	The references were added	Page 17<br>Line 13	One factor that contributes to patients' non-participation in the ward rounds is already known to be the complicated medical content [2, 25].
**#16** Pg 16, second paragraph: I find the use of the word "warm atmosphere" to be vague. Can you replace it with the specific aspects or contents that create the atmosphere conducive to patient participation and/or satisfaction?	We have erased this paragraph and explained "warm atmosphere" in Conclusions.	Page 20<br>Lines 2-7	**Please see the answer to the comment #20 of Reviewer 1.**
**#17** Pg 16, "The sampling in the study was stratified purposeful…" the rest of the sentence does not describe stratified purposeful (sometimes called stratified purposive) sampling, but rather a convenience sample. As noted above, the stratification scheme and<br>sampling method needs to be spelled out with greater detail. How many and what were the strata? How many individuals were sampled from each strata? From there, were	We have corrected: the sampling of the study was purposeful and captured information-rich cases to explore patients' experiences. Patients were selected with variation in age, gender and healthcare experience.<br>According to Patton (2015) convenience sampling is not purposeful.<br>Convenience sampling is based on the informants' availability, meaning "interviewing whoever happens to be at a place during a site visit" (Patton, 2015, p. 467). It was not the case of our study. In our study patients who have experience of ward rounds, are in different age and gender, were recruited.	Page 5<br>Lines 9-11<br><br>Page 18<br>Lines 5-6	**Please see the answer to the comment #8 of Reviewer1**<br><br>**We have revised as following:**<br><br>**Before:** The sampling in the study was stratified purposeful [22], meaning that informants who had experiences of ward rounds and were able to answer the research question were recruited.<br><br>**Now:** The sampling in the study was purposeful [22], meaning that informants who had experiences of ward rounds and were able to answer the research question were recruited

any adjustments made to ensure representation from each strata?	Even gatekeepers were used to select the information-rich cases to answer the study aim.		
**#18** Pg 17, first paragraph discussing the use of gatekeepers to identify patients: In methods, please describe gatekeepers with greater detail. In this limitations section, please describe how this could bias your results so that the reader can understand both the potential bias and why it was justified and make their own decisions regarding credibility.	We agree this need to be clearer.<br>We have described the gatekeepers in greater details in Participants/Sampling section as well as explained how the use of them could have influenced the results and how it was justified in Study Strengths and Limitations section.	Page 5 Lines 20-21<br><br>Page 18 Lines 6-14	**We have added the following sentence to Participants/Sampling:**<br>The gatekeepers were patient care coordinating nurses, who have the continuous and overall responsibility of a separate patient care, its planning, conducting, assessment and evaluation.<br><br>**We have revised as following:**<br><br>**Before:** Therefore, it was necessary to involve gatekeepers to identify patients, meaning that selection of patients to some extent rested on external third parties. This may affect credibility but also contributes to an increased possibility of reaching the desired population and capturing variations in age, ward experience, and gender.<br><br>**Now:** Therefore, it was necessary to involve gatekeepers to identify patients, meaning that selection of patients to some extent rested on external third parties. This may affect credibility due to pre-understanding of the gatekeepers. The patients considered not suitable by the gatekeepers were not asked to participate and, therefore, their experiences may not be shown in the findings. Still, as the gatekeepers were patientcare coordinating nurses who knew patients well, it contributed to patients' feeling comfortably to ask questions or to decline participation in the study as well as to an increased possibility of reaching the desired population and capturing variations in age, ward experience, and gender.
**#19** Pg 18, second paragraph: this fear of criticizing the hospital in which they were currently admitted is a significant limitation	Thank you for your remark. You are right that the fear of critisizing could influence the results towards patients' sharing more positive experiences. Still only some patients did not dare to	Page 19 Line 20	**We have addad the word "some" to the following sentence:**<br>To avoid destroying this good atmosphere, some patients did not dare criticize the ward rounds at the hospital where they were now, but they could do this when talking about their earlier experiences in other hospitals.

introduced by the decision to hold interviews while the patient was still admitted. Please include a discussion of how this could have biased results in this section. I understand that you have included it as part of your analysis, but it should also be detailed as a significant limitation.	critisiza, there were patients that openly and directly shared negative experiences. This could have shown the different personalities and behaviours of patients in general and their inclinations in communication with health professionals and could have been even manifested at the ward rounds themselves and lead to patients' involvement/uninvolvement. Morover, this reluctance was noticed and reported in the results, which shows that <u>all</u> results were repoprted which contributes to the credibility of the study.<br>Interviews were held while the patients was still admitted in hospital, which contributed to the fact that patients could share their freshly acquired experiences and not forget them.	Page 19 Lines 24-31<br><br>Page 19 Lines 29-31	**We have added following text to the last paragraph of Study Strengths and Limitations section:**<br><br>This could have influenced the results of the study by showing more positive patients' experiences. Still, there were patients who could criticize ward rounds. This reluctance of critique could reflect the behaviour and inclinations of patients in general and points out that more attention should be paid to it by healthcare professionals at ward rounds as well as communication should be adapted accordingly.<br><br>**We have put the following sentence to the end of the last paragraph of Strengths and Limitations section to make the text more coherent and clear:**<br><br>The fact that the interviews took place in close connection with rounds increased the possibility of capturing the patients' experiences and reduced the risk of impressions and feelings being forgotten.
**#20** Conclusions Pg 18, third paragraph: "The findings show that it is important to develop rounds in clinical settings," is vague. I'm not sure in what way you are recommending rounds to be developed.	We have modified the sentence and have written clearer.	Page 20 Lines 2-7	**The text was revised as follows:**<br><br>**Before:** The findings show that it is important to develop rounds in clinical settings and to create a model for this that can contribute to an open and supportive atmosphere and sharing of comprehensible and detailed information.<br><br>**After:** The findings show that it is important to develop a model of rounds in clinical settings which can contribute to an open and supportive atmosphere, when patients are invited into the dialogue, treated professionally and friendly, supported, given time and attention, and their feelings are respected. Patients experienced that sharing of comprehensible and detailed information in full as well as having opportunity to ask questions is important for them at ward rounds.

**#21** Figures<br>Figure 2 seems redundant with Table 1.	Thank you for the comment!<br>Table 1 and Figure 2 have different purposes: illustrating the analysis process and providing an overview of the results, correspondently.<br>Further, they differ in the contained information. Table 1 shows the process of analysis with compressed data extracts, whereas Figure 2 shows the results and includes all the codes, which is not true for Table 1, where just several codes are included. Figure 2 therefore, conveys all the results picture but has no data extracts.		
**Reviewer 2**			
**#1** Well written manuscript, clearly articulating the importance of ward rounds for patient care and patient centredness. The research question is very relevant and timely, addressing a gap in the international and Swedish context. The use of the explorative descriptive design with semi-structured interviews and reflexive thematic analysis is appropriate for the aim of investigating this theme. The study provides detailed patient quotes and nuanced insights into the diversity of patient experiences. I	We want to thank you for your positive feedback on our manuscript.		

quite like some statements such as "He took a chair and sat next to my bed. Just so there was some peace and quiet and that felt great. And then I felt what a difference it was to talk to him compared with the one standing by the door" which was a simple intervention with a profound effect on the perception of the patient.			
**#2** There are a few weak point such as sample size is small, homogenous (Swedish speaking) from a single hospital limiting external applicability.	We are in agreement with your comment and have already listed a limitation of Swedish-speaking homogenious group in Study Strengths and Limitations section in Discussion. Now we have also added a sentence about sample from a single hospital.<br>Though the interviews were conducted in a single hospital, different types of departments were included, such as medical and surgical to get some variation in the ward round work.	Page 18 Lines 17-25<br><br>Lines 20-22<br><br>Page 2	**The mentioned limitations were described in the following paragraph of Study Strengths and Limitations section:**<br><br>To participate in an interview, the patients needed to be verbally and cognitively capable and speak Swedish, which means that patients who could not take equally active roles in ward rounds due to, for example, language difficulties, cognitive function, or foreign origin did not have the opportunity to share their experiences. Further, participants were recruited from the single hospital, though from different departments: medical and surgical, to get the variation in the sample. The results can thus be seen as transferable to similar populations and contexts but might not apply to those not represented in the study. Transferability and confirmability were promoted through thick descriptions of the study population and context as well as data collection and analysis.<br><br>**The following sentence was added to this paragraph:**<br><br>Further, participants were recruited from the single hospital, though from different departments: medical and surgical, to get the variation in the sample.

	We have also added this limitation to Strengths and Limitations of the Study in Abstract.	Lines 5-6	**We have added the following limitation to Strengths and Limitations of the Study in Abstract:**<br><br>The sample was Swedish speaking patients from a single hospital which may limit transferability.
**#3** Gate keeper selection and patient`s reluctance to criticize may skew the findings towards more positive experience.	Please see the answers to komments #18 and #19 of Reviewer 1		
**#4** Why weren`t health care professional interviewed and were observes used to observe the discussions (Or are you planning to publish these later?).	Healthcare professionals' interviews as well as observations are planned to be conducted in further studies that investigate patient experiences and involvement in ward rounds. Moreover, patients' interviews could have given the insight what particulary should be in focus of interviews with personnel and obseravations.		
**#5 I**n conclusion, The study is a scientifically robust and societally relevant exploration of patients' experiences of ward rounds in Swedish inpatient care. I would like to commend authors for this relevant piece of work.	Thank you a lot for your positive conclusion!		
**Reviewer 3**			
**#1** Thank you for giving me the opportunity to discover this study. This topic is relevant for improving the shared decision-making strategy between patients and medical staff during	Thank you for your appreciative comment.<br>As for multidisciplinary approach when health professional team includes medical studets, the code ”many unknown people” covers patients' experiences of ward rounds when students are	Page 12-13<br>Lines 29-33; 1-12	Please see the text referring to the code ”many anknown people”

hospitalization. However, experiences can vary greatly depending on whether the medical team adopts a multidisciplinary approach, with or without the presence of numerous students. These considerations limit the data to medium-sized hospitals, as described in this study	present. And there patients even mention that unknown participants can destract their attention as well as if some participate for educational purposes, they should ask the patients' permission.		
**#2 Method:**<br>the interview guide seems appropriate. The authors appear to be well -trained in qualitative methods.	Thank you!		
**#3** Why did 20 patients decline to participate?	Now, we have explained it in the text.	Page 5, Line 25	**We have added the following sentence to Participants/Sampling:**<br>Other patients declined the participation due to health state or unwillingness.
**#4** Medical and surgical context: patients' experiences can vary considerably depending on the type of surgery or disease... It may be necessary to describe these contexts in more detail.	Though patients were hospitalized because of different reasons and had different health state during their state at hospital, still only those who were in the physical and mental condition to understand the information and give informed were asked to participate. Furthermore, those who felt that they would not be able to participate refused. Therefore, the part of the patients who had seriously bad health state had been not included. The results also show that patients' experiences depend on how the communication with healthcare professionals has been felt	Page 5 Lines 16-17<br><br>Lines 23-25	**We explain in following text:**<br>Patients who were not in the physical or mental condition to absorb information and give informed consent to participate were not asked.<br><br>In total, 36 participants were asked to participate in the study. Sixteen patients (nine women and seven men) accepted the invitation. The median age was 72 years (range 38–83 years). Other patients declined the participation due to health state or unwillingness.<br><br>**Also please see Findings section.**

	and if they have got answers to that they were wondering, not the disease or type of surgery. Though, impaired hearing and general condition could influence the communication.		
**#5** The patients' experiences could have been explored much more effectively using a GTA approach rather than a thematic analysis.	Reflexive thematic analysis is suitable for the study as according to experiential qualitative theoretical approach, language is viewed as a means of looking into patients' reality, which is socially dependent. Therefore the researcher and informants are believed to influence each other during the data collection and analysis. That is why reflexivity´, which is incorporated into Reflexive thematic analysis, was very important for the analysis as allowed the researchers to acknowledge their subjectivity and discuss preunderstandings. Grounded Theory Approach (GTA) was not used as its main focus is to develop a theory that explains certain process, which was not the aim of our research (Creswell & Poth, 2018). Morover, Grounded Theory is a suitable design when there are no suitable theories available or they do not cover the interested to the researcher variables (Creswell & Poth, 2018). But for determining which variables could be important/of interest, they should be explored and described first, which was focused upon in our study, as patients experiences of ward rounds need first be explored and described.		
**#6 Results:** easy to read, but too many comments from the authors, which should be shortened. More space should be given to verbatim quotes	We have shortened our comments where it could be possible without destroying the flow and the meaning.	Page 11<br><br>Page 11<br><br>Page 14	**We have erased following sentences:**<br><br>Daily contact and dialogues with their nurse contributed to the patients' sense of trust and they developed a closer relationship with the nurse.<br><br>The patients knew approximately what time the rounds usually took place and if the wait was too long, they would start to wonder.<br><br>Getting detailed information about their medical condition and results

			of examinations done and samples taken was important to the patients.
		Page 14	Lack of information about the risks of an operation could raise many concerns and getting clear and comprehensible information about the risks to oneself was highly desired. Patients said that the lack of information about the things they themselves were wondering about created a feeling of not being told and having to ask for information.
**#7** Very short interviews (median 15')... and only 16...	We agree, however, it is explained in Study Strengths and Limitations section in Discussion that interview length was regulated by patients and though they may be short their content was sufficient to answer the study's aim.	Page 19, Lines 4-6	**The following paragraph explains the interview length:**<br><br>Each interview's length was regulated by the patient. They could be considered short, which could influence the analysis due to the potential lack of data. However, the contents of the interviews were sufficient for the exploration of the patients' experiences of ward rounds.
**#8 Thematic analysis**: the least in-depth of the analytical prisms... (why not a GTA???)	Please, see answer to the comment **#5 of Reviewer 3.**<br><br>We believe, Thematic Analysis (TA) does not have the least depth among other analytical approaches. How deep it is depends on the aim of the study and data collected. The coding is situated on the continuuum between latent and manifest (Braun & Clarke, 2022). Still Braun and Clarke (2023) argue that shallow analysis with topic summary themes instead of meaning-based interpretive story is a common problem of the published articles that used TA. It may be the reason why TA is percieved as having the least depth. Here how they write about the depth of the TA: " The practice requires depth of engagement, thinking creatively and reflexively about the data, an intensive and organic coding process designed to parse out different facets of data meaning, and to help the researcher move beyond the most obvious or superficial meanings in the data." (Braun & Clarke, 2023, p. 3)		

**#9** So the range of results is limited: fig 2...	We are not sure that we understood the comment as the reviewer intended but we try to respond based on something that we think may refer to it. We assume that perhaps it is a matter of the introductory text in the Findings section stating that " The subthemes highlight factors that enable interaction between patients and healthcare professionals...". It might be understood that the results are limited to including interaction and not all possible experiences. We would like to admit that findings reflected in figure 2 include all experiences shared by patients, though these experiences encompass mostly interactions at the ward rounds.<br><br>We have revised the introductory text to make it clearer that figure 2 includes codes highlighting patients' experiences.	Page 9 Lines 28-32	**We have revised as following:**<br><br>**Before**:<br>The subthemes highlight factors that enable interaction between patients and healthcare professionals that can contribute to making the abstract and the inexplicable more concrete and comprehensible. The subthemes also highlight factors that make satisfactory interaction between patients and healthcare professionals impossible, causing the patient to feel uncertainty. Together, these subthemes illustrate the wide range of variation in patients' experience of ward rounds.<br><br>**Now:** The subthemes include codes highlighting patient's experiences of factors that enable interaction between patients and healthcare professionals that can contribute to making the abstract and the inexplicable more concrete and comprehensible. The subthemes also include codes highlighting patients' experiences of factors that make satisfactory interaction between patients and healthcare professionals impossible, causing the patient to feel uncertainty.
**#10** The limitations were very quickly listed in the discussion section...	Thank you for the remark. We have discussed some more limitations in Discussion section.		**Please see the answers to comments #18 and #19 of Reviewer 1**
**#11** The perspectives were also weak: continue to develop "an open and supportive atmosphere and sharing of comprehensible and detailed information"= Critical	We have explained in Conclusions what is meant by open and supportive atmosphere and sharing of comprehensive and detailed information.	Page 20 Lines 2-7	**We have revised as following:**<br><br>**Before:** The findings show that it is important to develop rounds in clinical settings and to create a model for this that can contribute to an open and supportive atmosphere and sharing of comprehensible and detailed information.<br><br>**Now:** The findings show that it is important to develop a model of rounds in clinical settings which

limits for the editorial board?			can contribute to an open and supportive atmosphere, when patients are invited into the dialogue, treated professionally and friendly, supported, given time and attention, and their feelings are respected. Patients experienced that sharing of comprehensible and detailed information in full as well as having opportunity to ask questions is important for them at ward rounds.

Braun, V., & Clarke, V. (2022). *Thematic Analysis. A Practical Guide*. Sage Publications Ltd.

Braun, V., & Clarke, V. (2023). Toward good practice in thematic analysis: Avoiding common problems and be(com)ing a knowing researcher. *Int J Transgend Health*, *24*(1), 1-6. https://doi.org/10.1080/26895269.2022.2129597

Creswell, J., & Poth, C. (2018). *Qualitative inquiry & research design. Choosing among five approaches* (4th ed.). Los Angeles: Sage Publications.

Heip, T., Van Hecke, A., Malfait, S., Van Biesen, W., & Eeckloo, K. (2022). The effects of interdisciplinary bedside rounds on patient centeredness, quality of care, and team collaboration: a systematic review. *Journal of patient safety*, *18*(1), e40-e44. https://doi.org/10.1097/pts.0000000000000695

Hunter, D., McCallum, J., & Howes, D. (2019). Defining exploratory-descriptive qualitative (EDQ) research and considering its application to healthcare. *Journal of Nursing and Health Care*, *4*(1).

Patton, M. (2015). *Qualitative Research and Evaluation Methods* (4nd ed.). Sage Publications.

Robinson, J. H., Callister, L. C., Berry, J. A., & Dearing, K. A. (2008). Patient-centered care and adherence: definitions and applications to improve outcomes. *Journal of the American Association of Nurse Practitioners*, *20*(12), 600-607. https://doi.org/10.1111/j.1745-7599.2008.00360.x

VERSION 2 - REVIEW

**Reviewer**	**1**
**Name**	**Vangompel , Emily White**
**Affiliation**	**University of Illinois Chicago**
**Date**	**15-Oct-2025**
**COI**	

All comments and concerns were well addressed.

**Reviewer**	**3**
**Name**	**PAUTRAT, Maxime**
**Affiliation**	**Université de Tours Faculté de Médecine**
**Date**	**13-Oct-2025**
**COI**	

Thank you for this second version and congratulations on your perseverance after all those initial comments.

Abstract: In the design section, it may be preferable to use "exploratory qualitative design" rather than "exploratory descriptive design."

Main text:

The authors have generally made an effort to respond to each of the comments made by the three reviewers. They defend their choice of reflexive thematic analysis (referring in particular to Braun et al.), and have added some details in the section on limitations concerning the small and homogeneous sample size and the brevity of the interviews.

I am not sure that a thematic analysis based on 16 short interviews allows for an in-depth exploration of the subject.

And even though it is important to conclude by recalling a model of medical visits with patients where "their feelings are respected," I still expect more than this obvious truth.

Finally, I note that my colleague reviewers were not bothered by these points, so I leave it to the editorial board to decide.

VERSION 2 - AUTHOR RESPONSE

**Reviewer 1**			
**#1** All comments and concerns were well addressed.	Thank you for yor valuable feedback on the manuscript.		
**Reviewer 3**			
**#1** Thank you for this second version and congratulations on your perseverance after all those initial comments.<br>**Abstract:** In the design section, it may be preferable to use "exploratory	Thank you for your appreciative comment.<br><br>We agree and have changed it.	Page 1 Line11	**We have revised as following:**<br>**Before:** An exploratory descriptive design was chosen**.**<br>**After:** An exploratory qualitative design was chosen

qualitative design" rather than "exploratory descriptive design."		Page 5 Lines 2	**Before:** An exploratory descriptive design was chosen to explore patients' experiences of ward rounds using semi-structured interviews [19, 20] and reflexive thematic analysis [21].<br>**Adfter:** An exploratory qualitative design was chosen to explore patients' experiences of ward rounds using semi-structured interviews [19, 20] and reflexive thematic analysis [21].
**#2 Main Text:**<br>The authors have generally made an effort to respond to each of the comments made by the three reviewers. They defend their choice of reflexive thematic analysis (referring in particular to Braun et al.) and have added some details in the section on limitations concerning the small and homogeneous sample size and the brevity of the interviews.	Thank you for your feedback!		
**#3** I am not sure that a thematic analysis based on 16 short interviews allows for an in-depth exploration of the subject.	The study findings show that the data were rich and diverse in meaning. This is seen in two subthemes that show a wide variety and a contradiction in patients' experiences. And according to Braun and Clarke (2022), quality of data and information power, meaning richness and complexity of data, matters. Not precise calculations.<br>Moreover, patients regulated the time and extent of their answers, which was respected, taking into account		

	possible patients' vulnerability.<br>We believe that the quality and richness of data does not depend on how much participants say, but on what they say and what is the meaning of it.		
**#4** And even though it is important to conclude by recalling a model of medical visits with patients where "their feelings are respected," I still expect more than this obvious truth.<br>Finally, I note that my colleague reviewers were not bothered by these points, so I leave it to the editorial board to decide.	Even if it may seem obvious that a model of rounds should be created, where patients' feelings are respected, still, our study shows that it is not always like this in real life. Therefore, we mention it in conclusions as a recommendation for clinical practise. Moreover, this statement concerns patients' feelings, which is not so obvious, as the round definition and structure includes mostly treatment and care planning as well as information provision to patients, but does not take into account their emotional state (Heip et al., 2022; O'Hare, 2008).<br>There were other findings in our study, except the need to respect patients' feelings, which hopefully could meet your expectations. The findings are divided into 2 subthemes: feeling of togetherness versus loneliness and getting answers versus being left in limbo, which include codes provided in Figure 2.<br>There is also a finding that differs from other research, which is mentioned in Discussion, second paragraph. It is about that patients did not feel a lack of time during	Page 9-15<br><br>Page 15-16<br>Lines 31-36; 1-10	**The text in the manuscript:**<br>This can explain several paradoxes shown in the findings, which, to our knowledge, have not been shown in other research. First, patients feel

	the rounds and other factors influenced their experiences, such as trust to healthcare professionals and satisfaction with their care, which, paradoxically, can lead even to lower patients' participation. Further we write about several paradoxes in our findings, which, we believe are not obvious.		disadvantaged when they cannot participate in decision-making, unless they feel such trust for healthcare professionals that they can hand over decision-making to them. Second, patients do not dare criticize professionals even if they do not receive all the necessary information, as they are afraid of ruining their relationships with the professionals.

Braun, V., & Clarke, V. (2022). *Thematic Analysis. A Practical Guide*. Sage Publications Ltd.

Heip, T., Van Hecke, A., Malfait, S., Van Biesen, W., & Eeckloo, K. (2022). The effects of interdisciplinary bedside rounds on patient centeredness, quality of care, and team collaboration: a systematic review. *Journal of patient safety*, *18*(1), e40-e44. https://doi.org/10.1097/pts.0000000000000695

O'Hare, J. A. (2008). Anatomy of the ward round. *Eur J Intern Med*, *19*(5), 309-313. https://doi.org/10.1016/j.ejim.2007.09.016
